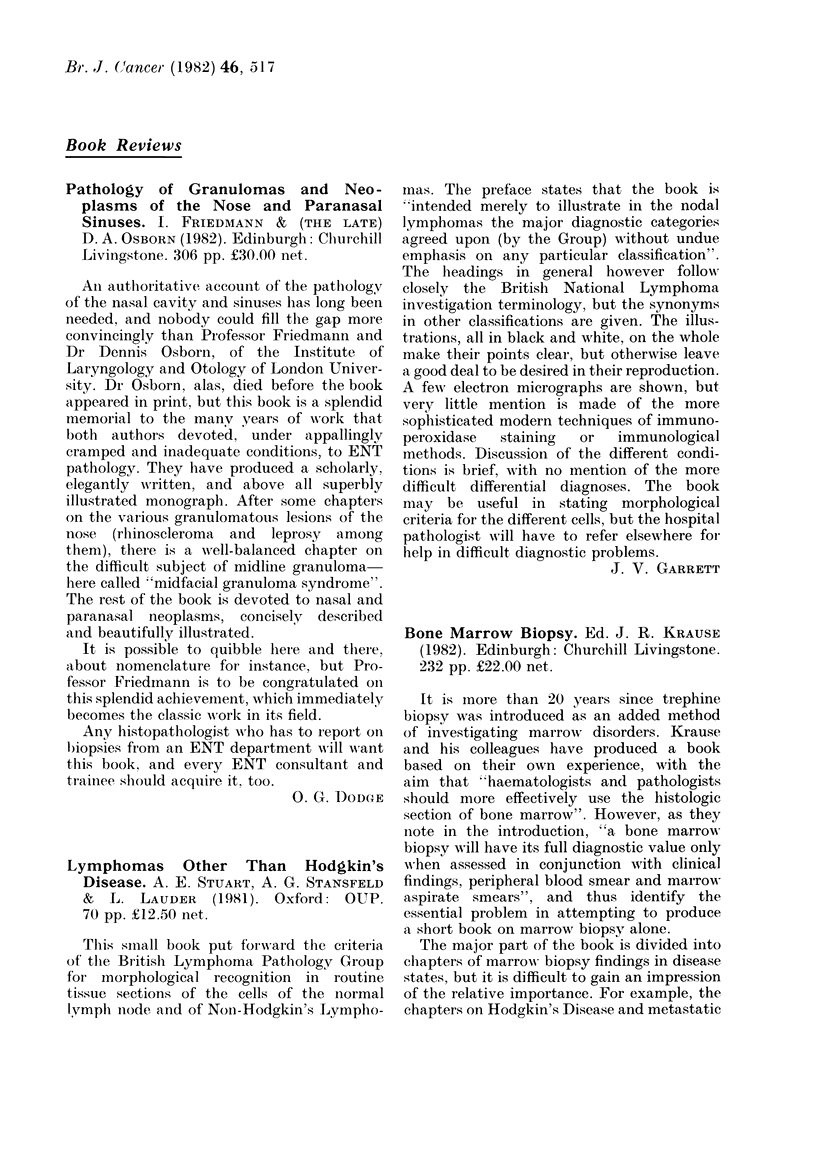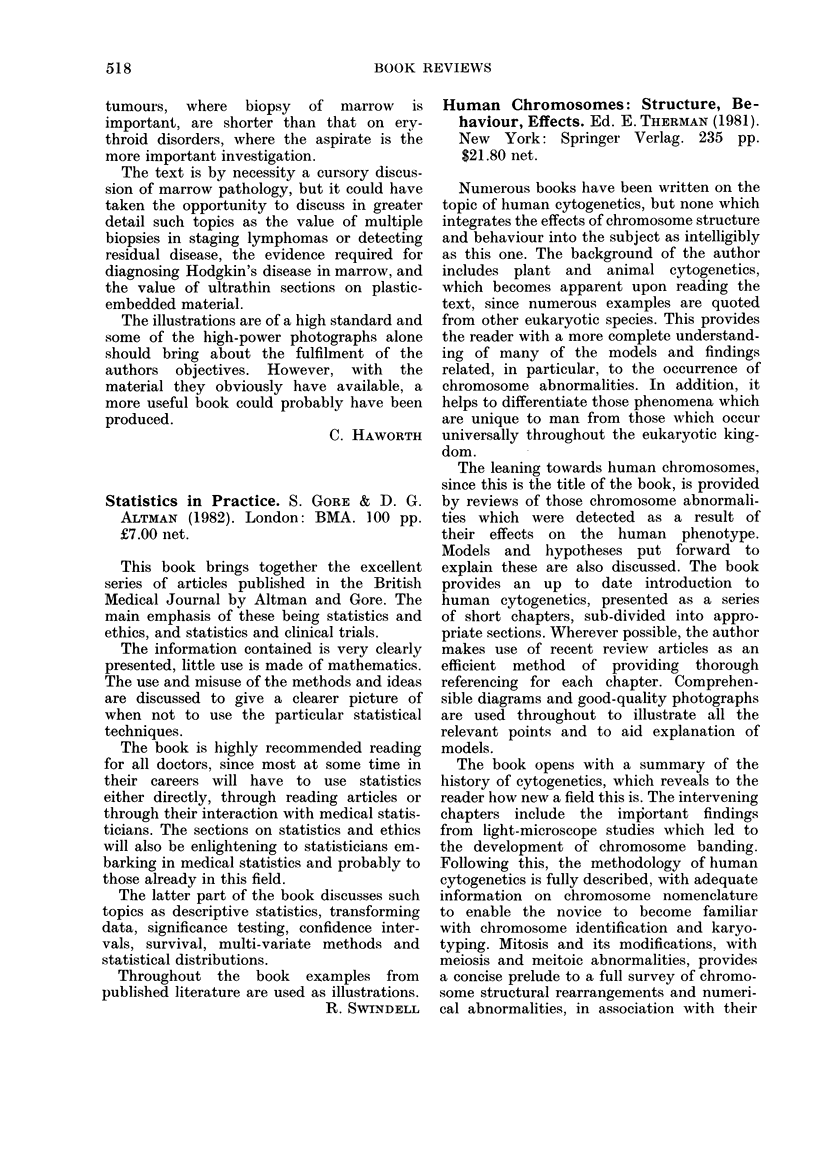# Bone Marrow Biopsy

**Published:** 1982-09

**Authors:** C. Haworth


**Bone Marrow Biopsy.** Ed. J. R. Krause (1982). Edinburgh: Churchill Livingstone. 232 pp. £22.00 net.

It is more than 20 years since trephine biopsy was introduced as an added method of investigating marrow disorders. Krause and his colleagues have produced a book based on their own experience, with the aim that "haematologists and pathologists should more effectively use the histologic section of bone marrow". However, as they note in the introduction, "a bone marrow biopsy will have its full diagnostic value only when assessed in conjunction with clinical findings, peripheral blood smear and marrow aspirate smears", and thus identify the essential problem in attempting to produce a short book on marrow biopsy alone.

The major part of the book is divided into chapters of marrow biopsy findings in disease states, but it is difficult to gain an impression of the relative importance. For example, the chapters on Hodgkin's Disease and metastatic tumours, where biopsy of marrow is important, are shorter than that on erythroid disorders, where the aspirate is the more important investigation.

The text is by necessity a cursory discussion of marrow pathology, but it could have taken the opportunity to discuss in greater detail such topics as the value of multiple biopsies in staging lymphomas or detecting residual disease, the evidence required for diagnosing Hodgkin's disease in marrow, and the value of ultrathin sections on plastic-embedded material.

The illustrations are of a high standard and some of the high-power photographs alone should bring about the fulfilment of the authors objectives. However, with the material they obviously have available, a more useful book could probably have been produced.

C. HAWORTH